# Topology degree results on a G-ABC implicit fractional differential equation under three-point boundary conditions

**DOI:** 10.1371/journal.pone.0300590

**Published:** 2024-07-01

**Authors:** Shahram Rezapour, Sabri T. M. Thabet, Ava Sh. Rafeeq, Imed Kedim, Miguel Vivas-Cortez, Nasser Aghazadeh

**Affiliations:** 1 Institute of Research and Development, Duy Tan University, Da Nang, Vietnam; 2 Department of Mathematics, Azarbaijan Shahid Madani University, Tabriz, Iran; 3 Insurance Research Center (IRC), Tehran, Iran; 4 Department of Medical Research, China Medical University Hospital, China Medical University,Taichung, Taiwan; 5 Department of Mathematics, Saveetha School of Engineering, Saveetha Institute of Medical and Technical Sciences, Saveetha University, Chennai, Tamil Nadu, India; 6 Department of Mathematics, Radfan University College, University of Lahej, Lahej, Yemen; 7 Department of Mathematics, College of Science, Korea University, Seoul, South Korea; 8 Department of Mathematics, College of Science, University of Zakho, Duhok, Iraq; 9 Department of Mathematics, College of Science and Humanities in Al-Kharj, Prince Sattam Bin Abdulaziz University, Al-Kharj, Saudi Arabia; 10 Faculty of Exact and Natural Sciences, School of Physical Sciences and Mathematics, Pontifical Catholic University of Ecuador, Sede Quito, Ecuador; 11 Department of Mathematics, Izmir Institute of Technology, Izmir, Türkiye; University of Education, PAKISTAN

## Abstract

This research manuscript aims to study a novel implicit differential equation in the non-singular fractional derivatives sense, namely Atangana-Baleanu-Caputo (ABC) of arbitrary orders belonging to the interval (2, 3] with respect to another positive and increasing function. The major results of the existence and uniqueness are investigated by utilizing the Banach and topology degree theorems. The stability of the Ulam-Hyers (UH) type is analyzed by employing the topics of nonlinear analysis. Finally, two examples are constructed and enhanced with some special cases as well as illustrative graphics for checking the influence of major outcomes.

## 1 Introduction

The derivatives of non-integer orders, or fractional derivatives, are a mathematical concept that extends differentiation beyond integer orders. These types of derivatives have a large domain of applications in numerous fields, including physics, engineering, and finance, for additional details see these manuscripts [1–8]. In the realm of fractional calculus has seen the advent of a fresh fractional operator by Atangana and Baleanu (AB) [9], which is free from singularity kernels. This operator is established through the Mittag-Lefler function in the Caputo and Riemann-Liouville contexts. The non-singular fractional operators are well-behaved and allow for more accurate modeling of the underlying system. The AB operator can be used to describe phenomena such as diffusion, wave propagation, and viscoelasticity, and has applications in many fields such as image processing, signal analysis, and control theory. Non-singular fractional operators are important tools for researchers and practitioners seeking to understand and manipulate complex systems in a variety of contexts and they have stimulated a great deal of interest among researchers in their applicability to diverse problems, we indicate the readers to these works [10–19] and the references therein. Subsequently, authors of this work [20] popularized the AB definition to contain differentiation and integration with respect to non-negative, non-decreasing function, leading to the development of the G-AB operator. In 2023, Abdeljawad et al. [21] expanded this operator to higher-order fractional derivatives and integrals. Furthermore, this type of fractional derivative is a generalization of the traditional derivative, where the derivative is taken with respect to a function rather than a variable. This type of derivative is commonly used in fractional calculus with various operators to describe complex systems with non-integer order dynamics. In fact, by taking this fractional derivative, the researchers can better model the behavior of these systems and gain a deeper understanding of their underlying dynamics, see for example [22–24] and references cited therein.

An implicit differential equation involving a fractional derivative of an unknown function that appears implicitly in the equation has several benefits, including the ability to model complex systems with memory effects, non-local interactions, and anomalous diffusion. These equations also accurately describe physical phenomena such as transport in porous media or viscoelastic materials [25–28]. In particular, Thabet and Kedim [29] studied a Hilfer fractional snap dynamic system on an infinite interval. Authors [30] discussed stability analysis of fractional pantograph implicit differential equations with initial boundary and impulsive conditions. Also, AB fractional derivative used to investigate the stability of implicit differential problem by authors [31].

Recently in 2022, Shah et al. [32] used degree theory to establish qualitative results for the following differential equation:

$$\begin{array}{c} \left\{ \begin{array}{c} y^{\prime \prime \prime }\left(\upsilon \right)+\varpi (\upsilon ,\text{y}(\upsilon ))=0,\upsilon \in \text{J}=\left[\iota ,\rho \right],\\ \text{y}\left(\iota \right)=0,\;{\text{y}}^{\prime }\left(\iota \right)=0,\;\text{y}\left(\rho \right)=\xi \text{y}\left(s\right),\;s\in \left(\iota ,\rho \right). \end{array}\right. \end{array}$$ (1.1)

Very recently, authors [33] extended the above equation (1.1) to the Caputo fractional order derivative for discussing the qualitative results and some types of UH stability as in the following form:

{DCιμy(υ)+ϖ(υ,y(υ))=0,υ∈J=[ι,ρ],μ∈(2,3],y(ι)=0,y′(ι)=0,y(ρ)=ξy(s),s∈(ι,ρ). (1.2)

Inspired by the research mentioned above articles, in this current work, we study the qualitative properties of the solution for G-ABC implicit fractional differential equation (**IFDE**) of the following form:

{DABCιμ,Gy(υ)=ϖ(υ,y(υ),DABCιμ,Gy(υ)),υ∈J=[ι,ρ],y(ι)=0,[y(ι)]G′=0,y(ρ)=ξy(s),s∈(ι,ρ), (1.3)

where DABCιμ,G denotes the G-ABC fractional derivatives of arbitrary order *μ* ∈ (2, 3] and the function $\varpi :\text{J}\times R^{2}\to R$ is continuous. Additionally, $G:\left[\iota ,\rho \right]\to R^{+}$ be a non-decreasing and non-negative function with $G\left(\upsilon \right)\in C^{1}\left(\left(\iota ,\rho \right),R^{+}\right)$, such that $[\text{y}(\upsilon ){]}_{G}^{\prime }=(\frac{1}{G^{\prime }\left(\upsilon \right)}\frac{d}{d\upsilon })\text{y}\left(\upsilon \right)$ and $G^{\prime }\left(\upsilon \right)\neq 0,\;\forall \;\upsilon \in \text{J}.$ Furthermore, $(G(\rho )-G(\iota ){)}^{2}\neq \xi (G(s)-G(\iota ){)}^{2},$ and ξ∈R.

In this situation, we would like to indicate that our contributions are interesting and the Eq (1.3) is new in the framework of G-ABC fractional order derivatives which include ABC derivative as a special case when G(υ)=υ. Moreover, an approach analysis in this work is different about methods used in these works [32, 33], and the Eq (1.3) covers many problems available in the literature studies, for instance,

(**i**) the Eq (1.3) reduces to problem (1.1) if *μ* → 3, and the implicit term omitted;

(**ii**) the Eq (1.3) returns to problem (1.2) if we replace the operator DABCιμ,G by DCιμ with omitting the implicit term.

The remaining parts of this paper are arranged as follows: Sec.2 is devoted to recalling the basic background materials related to fractional calculus and nonlinear analysis. Sec.3 discusses the existence and uniqueness theorems by using FPTs. Sec.4 is investigated UH stability. Finally, Sec.5 is dedicated to testing the effectiveness of main outcomes.

## 2 Preliminaries

In this situation, we present essential background material. Consider the space of continuous functions denoted by Ω≔C(J=[ι,ρ],R) which is Banach space gifted with the norm ‖y‖ = sup_*υ*∈[*ι*,*ρ*]_|y(*υ*)|.

**Definition 2.1** [34] *Let* y:[ι,ρ]→R *and μ* > 0, *then the equation*

IRLιμ,Gy(υ)=1Γ(μ)∫ιυ(G(υ)-G(z))μ-1G′(z)y(z)dz,

*is μ*^*th*^ *order of* G-*Riemann–Liouville fractional integral, where* Γ *is Gamma function*.

**Definition 2.2** [21]. *The* G-ABC *fractional derivatives of* ${\text{y}}^{\left(m+1\right)}\in H^{1}\left(\iota ,\rho \right)$ *with order μ* ∈ (*m*, *m* + 1], *ν* = *μ* − *m*, *m* = 0, 1, 2, …, *is defined as*

DABCιμ,Gyυ=DABCιν,GyGmυ=Φμ−mm+1−μ∫ιυG′zEμ−m−μ−mm+1−μGυ−Gzμ−myGm+1zdz,

*where* ${\text{y}}_{G}^{\left(m\right)}\left(\upsilon \right)=(\frac{1}{G^{\prime }\left(\upsilon \right)}\frac{d}{d\upsilon }{)}^{m}\text{y}\left(\upsilon \right)$ *and* ${\text{y}}_{G}^{\left(0\right)}\left(\upsilon \right)=\text{y}\left(\upsilon \right)$. *If* μ=k∈N, *then* (DABCιμ,Gy)(υ)=yG(k)(υ). *Furthermore*, $E_{\mu }$ *is the Mittag-Leffler function*

$$\begin{array}{c} E_{\mu }(\text{x})=\sum_{j=0}^{\infty }\frac{{\text{x}}^{j}}{\Gamma (\mu j+1)},\;Re(\mu )>0,\text{x}\in C, \end{array}$$

*and* Φ(*μ*) *denotes the normalization function endowed by* Φ(0) = Φ(1) = 1.

**Definition 2.3** [21]. *The following relation*:

(IABιμ,Gy)υ=IRLιm,GIABιv,Gyυ=IABιv,GIRLιm,Gyυ=m+1−μΦμ−mIRLιm,Gyυ+μ−mΦμ−mIRLιμ,Gyυ,

*is* G-AB *fractional integral of a function* y *with order μ* ∈ (*m*, *m* + 1], *ν* = *μ* − *m*, *m* = 0, 1, 2, …, *where* ℑRLιm,G *is* G-*Riemann–Liouville fractional integral*.

**Lemma 2.4** [21]. *For μ* ∈ (*m*, *m* + 1], *ν* = *μ* − *m*, *m* = 0, 1, 2, …, *and* $\text{y}\in H^{1}\left(\text{J},R\right),$ *and* $G\in C^{m}\left(\text{J},R\right)$. *Then*

IABιμ,GDABCιμ,Gy(υ)=y(υ)-∑r=0mdr(G(υ)-G(ι))r,dr∈R.

**Lemma 2.5** [21]. *For μ* ∈ (*m*, *m* + 1], *ν* = *μ* − *m*, *m* = 0, 1, 2, …, *ϵ* > 0, *and* $G\in C^{m}\left(\text{J},R\right),$ *with* $G^{\prime }\left(\upsilon \right)\neq 0.$ *Then*,

*(i)* IABιμ,G[G(υ)-G(ι)]ϵ=(m+1-μ)Γ(ϵ+1)[G(υ)-G(ι)]ϵ+mΦ(μ-m)Γ(m+ϵ+1)+(μ-m)Γ(ϵ+1)[G(υ)-G(ι)]ϵ+μΦ(μ-m)Γ(μ+ϵ+1) ;
*(ii)* (IABιμ,G1)(υ)=(m+1-μ)[G(υ)-G(ι)]mΦ(μ-m)Γ(m+1)+(μ-m)[G(υ)-G(ι)]μΦ(μ-m)Γ(μ+1) .

Now, we about to introduce a definition of the Kuratowski’s measure of noncompactness *χ*(⋅) as follows:

$$\begin{array}{c} \chi \left(F\right)=\text{inf}\{\varepsilon >0:\;F=\cup_{i=1}^{n}F_{i}\;\text{and}\;diam\left(F_{i}\right)\leq \varepsilon ,n\in N\}, \end{array}$$

where $diam\left(F_{i}\right)=sup\{|\text{y}-\hat{\text{y}}\left|:\text{y},\hat{\text{y}}\in F_{i}\right\}$, and F is a bounded subset of the Banach space Ω. It is clear that 0≤χ(F)≤diam(F)<+∞ [35].

**Definition 2.6** [35] *Let* D:N→H *be bounded and continuous with* N⊂H. *Then*, D *will be χ-Lipschitz if* ∃ *ϵ* ≥ 0, *so that*

$$\begin{array}{c} \chi (D(B))<\epsilon \chi (B),\;\forall \;bounded\;B\subset N. \end{array}$$

*As well as*, D *is named as strict χ*-*contraction when ϵ* < 1 *holds*.

**Definition 2.7** [35] *A function* D *is χ-condensing if*

$$\begin{array}{c} \chi (D(B))<\chi (B),\forall B\subset N\;bounded,\;\text{with}\;\chi (B)>0. \end{array}$$

*So*, χ(D(B))≥χ(B) *gives χ*(*B*) = 0. *Also*, D:N→H *is Lipschitz for ϵ* > 0 *such that*

$$\begin{array}{c} \|D\left(\text{y}\right)-D\left(\hat{\text{y}}\right)\|\leq \epsilon \|\text{y}-\hat{\text{y}}\|\;for\;all\;\text{y},\hat{\text{y}}\in N. \end{array}$$

*If ϵ* < 1, *in this case* D *is called a strict contraction*.

**Lemma 2.8** [35] D *is χ-Lipschitz with constant ϵ* = 0 *iff* D:N→H *is compact*.

**Lemma 2.9** [35] *A function* D *is χ-Lipschitz with constant ϵ iff* D:N→H *is Lipschitz with Lipschitz constant ϵ*.

**Theorem 2.10** [36] *Let* D:Ω→Ω *be an χ-condensing and*

$$\begin{array}{c} W=\{\text{y}\in \Omega :\;\zeta \in [0,1]\;exists\;so\;that\;\text{y}=\zeta D(\text{y})\}. \end{array}$$

*If* W *is a bounded subset contained in* Ω, *i.e., a constant k* > 0 *exists with* $W\subset B_{k}\left(0\right),$ *then* $deg(I-\zeta D,B_{k}\left(0\right),0)=1$ *for all ζ* ∈ [0, 1]. *Therefore*, D *has a fixed point and the set* FIX(D) *belongs to B*_*k*_(0).

## 3 Existence and uniqueness analysis

We introduce an equivalent integral fractional equation of the G-ABC **IFDE** (1.3). Regarding this, we first derive the following lemma:

**Lemma 3.1** *Let* $\mu \in \left(2,3\right],,{\text{y}}^{\left(3\right)}\in H^{1}\left(\iota ,\rho \right),q\in \Omega$ *and* $Z=(G(\rho )-G(\iota ){)}^{2}-\xi (G(s)-G(\iota ){)}^{2}\neq 0$ . *Then, the* G-ABC *fractional differential problem*:

{DABCιμ,Gy(υ)=q(υ),υ∈J=[ι,ρ],μ∈(2,3],y(ι)=0,[y(ι)]G′=0,y(ρ)=ξy(s),s∈(ι,ρ), (3.1)

*is equivalent to*

yυ=ξ(Gυ−Gι)2ZIABιμ,Gqs−Gυ−Gι2ZIABιμ,Gqρ+IABιμ,Gqυ.

**Proof** At the beginning, we apply IABιμ,G on both sides of Eq (3.1) and using Lemma 2.4, we get

y(υ)=e0+e1(G(υ)-G(ι))+e2(G(υ)-G(ι))2+IABιμ,Gq(υ)=e0+e1(G(υ)-G(ι))+e2(G(υ)-G(ι))2+3-μΦ(μ-2)IRLι2,Gq(υ)+(μ-2)Φ(μ-2)IRLιμ,Gq(υ).

So, due to the condition y(*ι*) = 0, we deduce that *e*_0_ = 0. Thus, by substituting the value of *e*_0_ and by taking the first derivative with respect to a function G, we find

[y(υ)]G′=e1+2e2(G(υ)-G(ι))+3-μΦ(μ-2)IRLι1,Gq(υ)+(μ-2)Φ(μ-2)IRLιμ-1,Gq(υ),

and due to the boundary condition ${\left[\text{y}\left(\iota \right)\right]}_{G}^{\prime }=0$, we have *e*_1_ = 0, which yields that

y(υ)=e2(G(υ)-G(ι))2+3-μΦ(μ-2)IRLι2,Gq(υ)+(μ-2)Φ(μ-2)IRLιμ,Gq(υ).

Next, by applying the condition y(ρ)=ξy(s), one has

e2(G(ρ)-G(ι))2+IABιμ,Gq(ρ)=e2ξ(G(s)-G(ι))2+ξIABιμ,Gq(s),

which implies that

e2=1Z[ξIABιμ,Gq(s)-IABιμ,Gq(ρ)].

Hence, we deduce that

y(υ)=(G(υ)-G(ι))2Z[ξIABιμ,Gq(s)-IABιμ,Gq(ρ)]+IABιμ,Gq(υ)=ξ(G(υ)-G(ι))2ZIABιμ,Gq(s)-(G(υ)-G(ι))2ZIABιμ,Gq(ρ)+IABιμ,Gq(υ).

Therefore, the proof is finished.

As a consequence of the above lemma, we present the following essential result:

**Lemma 3.2** *Let* $\mu \in \left(2,3\right],{\text{y}}^{\left(3\right)}\in H^{1}\left(\iota ,\rho \right)$ *and* $Z=(G(\rho )-G(\iota ){)}^{2}-\xi (G(s)-G(\iota ){)}^{2}\neq 0$ . *Then, the* G-ABC **IFDE** (1.3) *has a solution equivalent to*

y(υ)=ξ(G(υ)-G(ι))2ZIABιμ,Gϖ(s,y(s),DABCιμ,Gy(s))-(G(υ)-G(ι))2ZIABιμ,Gϖ(ρ,y(ρ),DABCιμ,Gy(ρ))+IABιμ,Gϖ(υ,y(υ),DABCιμ,Gy(υ)). (3.2)

Now, to achieve the required existence and uniqueness theorems, according to Lemma 3.2, the solution of the G-ABC **IFDE** (1.3) is a fixed point of the operator ℵ: Ω → Ω which is defined as:

(ℵy)(υ)=ξ(G(υ)-G(ι))2ZIABιμ,Gϖ(s,y(s),DABCιμ,Gy(s))-(G(υ)-G(ι))2ZIABιμ,Gϖ(ρ,y(ρ),DABCιμ,Gy(ρ))+IABιμ,Gϖ(υ,y(υ),DABCιμ,Gy(υ)). (3.3)

For working analysis, we state the following conditions:

(*AS*_1_) There are the constants *δ*_1_, *δ*_2_, *δ*_3_ > 0, such that
  $$\begin{array}{c} |\varpi (\upsilon ,\text{y}\left(\upsilon \right),\hat{\text{y}}\left(\upsilon \right))|\leq \delta_{1}+\delta_{2}\left|\text{y}\left(\upsilon \right)\right|+\delta_{3}\left|\hat{\text{y}}\left(\upsilon \right)\right|, \end{array}$$
  for any $\text{y},\hat{\text{y}}\in \Omega$ and *υ* ∈ J.
(*AS*_2_) There are the constants *ℓ*_1_ > 0 and *ℓ*_2_ ∈ (0, 1), for any ${\text{y}}_{1},{\text{y}}_{2},{\hat{\text{y}}}_{1},{\hat{\text{y}}}_{2}\in \Omega$ and *υ* ∈ J, satisfy
  $$\begin{array}{c} |\varpi (\upsilon ,{\text{y}}_{1},{\text{y}}_{2})-\varpi (\upsilon ,{\hat{\text{y}}}_{1},{\hat{\text{y}}}_{2})|\leq \ell_{1}|{\text{y}}_{1}-{\text{y}}_{2}|+\ell_{2}\left|{\hat{\text{y}}}_{1}-{\hat{\text{y}}}_{2}\right|. \end{array}$$

For simplicity, we set

$$\begin{array}{cc} \Pi_{1} & =\frac{\left|\xi \right|{\left(G(\rho )-G(\iota )\right)}^{2}}{\left|Z\right|}\frac{\delta_{1}}{1-\delta_{3}}[\frac{\left(3-\mu \right){\left[G\left(s\right)-G\left(\iota \right)\right]}^{2}}{\Phi (\mu -2)\Gamma (3)}+\frac{\left(\mu -2\right){\left[G\left(s\right)-G\left(\iota \right)\right]}^{\mu }}{\Phi (\mu -2)\Gamma (\mu +1)}]\\ & \;+[\frac{{\left(G(\rho )-G(\iota )\right)}^{2}}{\left|Z\right|}+1]\frac{\delta_{1}}{1-\delta_{3}}[\frac{\left(3-\mu \right){\left[G\left(\rho \right)-G\left(\iota \right)\right]}^{2}}{\Phi (\mu -2)\Gamma (3)}+\frac{\left(\mu -2\right){\left[G\left(\rho \right)-G\left(\iota \right)\right]}^{\mu }}{\Phi (\mu -2)\Gamma (\mu +1)}];\\ \Pi_{2} & =\frac{\left|\xi \right|{\left(G(\rho )-G(\iota )\right)}^{2}}{\left|Z\right|}\frac{\delta_{2}}{1-\delta_{3}}[\frac{\left(3-\mu \right){\left[G\left(s\right)-G\left(\iota \right)\right]}^{2}}{\Phi (\mu -2)\Gamma (3)}+\frac{\left(\mu -2\right){\left[G\left(s\right)-G\left(\iota \right)\right]}^{\mu }}{\Phi (\mu -2)\Gamma (\mu +1)}]\\ & \;+[\frac{{\left(G(\rho )-G(\iota )\right)}^{2}}{\left|Z\right|}+1]\frac{\delta_{2}}{1-\delta_{3}}[\frac{\left(3-\mu \right){\left[G\left(\rho \right)-G\left(\iota \right)\right]}^{2}}{\Phi (\mu -2)\Gamma (3)}+\frac{\left(\mu -2\right){\left[G\left(\rho \right)-G\left(\iota \right)\right]}^{\mu }}{\Phi (\mu -2)\Gamma (\mu +1)}]. \end{array}$$

**Theorem 3.3** *Under assumption* (*AS*_1_) *with δ*_3_ ≠ 1. *The mapping* ℵ: Ω → Ω *is continuous and satisfy the growth condition* ‖ℵy‖ ≤ Π_1_ + Π_2_‖y‖.

**Proof** We define a bounded ball $D_{r}$ as $D_{r}=\left\{\text{y}\in \Omega :\|\text{y}\|\leq r\right\}$. Regarding to show the continuity of ℵ, let us taking the convergence sequence ${\left\{{\text{y}}_{n}\right\}}_{n\in N}$ to y in the ball ℧_*ς*_ as *n* → ∞. Thus, by continuity of *ϖ* and by applying Lebesgue dominated convergence theorem, one has

limn→∞(ℵyn)(υ)=ξ(G(υ)-G(ι))2ZIABιμ,Glimn→∞ϖ(s,yn(s),DABCιμ,Gyn(s))-(G(υ)-G(ι))2ZIABιμ,Glimn→∞ϖ(ρ,yn(ρ),DABCιμ,Gyn(ρ))+IABιμ,Glimn→∞ϖ(υ,yn(υ),DABCιμ,Gyn(υ))=(ℵy)(υ).

Hence, ℵ is continuous.

Next, regarding to the growth condition, by applying (*AS*_1_), we find

|(ℵy)(υ)|≤|ξ|(G(υ)-G(ι))2|Z|IABιμ,G|ϖ(s,y(s),DABCιμ,Gy(s))|+(G(υ)-G(ι))2|Z|IABιμ,G|ϖ(ρ,y(ρ),DABCιμ,Gy(ρ))|+IABιμ,G|ϖ(υ,y(υ),DABCιμ,Gy(υ))|≤|ξ|(G(υ)-G(ι))2|Z|IABιμ,G(δ1+δ2|y(s)|+δ3|DABCιμ,Gy(s)|)+(G(υ)-G(ι))2|Z|IABιμ,G(δ1+δ2|y(ρ)|+δ3|DABCιμ,Gy(ρ)|)+IABιμ,G(δ1+δ2|y(υ)|+δ3|DABCιμ,Gy(υ)|). (3.4)

Since, DABCιμ,Gy(υ)=ϖ(υ,y(υ),DABCιμ,Gy(υ)), then

|DABCιμ,Gy(υ)|≤δ1+δ2|y(υ)|+δ3|DABCιμ,Gy(υ)|,

which implies that

|DABCιμ,Gy(υ)|≤δ11-δ3+δ21-δ3|y(υ)|. (3.5)

Therefore, in view of the Eqs (3.4) and (3.5), and taking supremum, one has

$$\begin{array}{cc} & \left\|ℵ\text{y}\right\|\\ & \leq \frac{\left|\xi \right|{\left(G\left(\rho \right)-G\left(\iota \right)\right)}^{2}}{\left|Z\right|}\left(\frac{\delta_{1}}{1-\delta_{3}}+\frac{\delta_{2}}{1-\delta_{3}}\left\|\text{y}\right\|\right)[\frac{\left(3-\mu \right){\left[G\left(s\right)-G\left(\iota \right)\right]}^{2}}{\Phi \left(\mu -2\right)\Gamma \left(3\right)}+\frac{\left(\mu -2\right){\left[G\left(s\right)-G\left(\iota \right)\right]}^{\mu }}{\Phi \left(\mu -2\right)\Gamma \left(\mu +1\right)}]\\ & \;+\frac{{\left(G\left(\rho \right)-G\left(\iota \right)\right)}^{2}}{\left|Z\right|}\left(\frac{\delta_{1}}{1-\delta_{3}}+\frac{\delta_{2}}{1-\delta_{3}}\left\|\text{y}\right\|\right)[\frac{\left(3-\mu \right){\left[G\left(\rho \right)-G\left(\iota \right)\right]}^{2}}{\Phi \left(\mu -2\right)\Gamma \left(3\right)}+\frac{\left(\mu -2\right){\left[G\left(\rho \right)-G\left(\iota \right)\right]}^{\mu }}{\Phi \left(\mu -2\right)\Gamma \left(\mu +1\right)}]\\ & \;+\left(\frac{\delta_{1}}{1-\delta_{3}}+\frac{\delta_{2}}{1-\delta_{3}}\left\|\text{y}\right\|\right)[\frac{\left(3-\mu \right){\left[G\left(\rho \right)-G\left(\iota \right)\right]}^{2}}{\Phi \left(\mu -2\right)\Gamma \left(3\right)}+\frac{\left(\mu -2\right){\left[G\left(\rho \right)-G\left(\iota \right)\right]}^{\mu }}{\Phi \left(\mu -2\right)\Gamma \left(\mu +1\right)}]\\ & \leq \frac{\left|\xi \right|{\left(G\left(\rho \right)-G\left(\iota \right)\right)}^{2}}{\left|Z\right|}\frac{\delta_{1}}{1-\delta_{3}}[\frac{\left(3-\mu \right){\left[G\left(s\right)-G\left(\iota \right)\right]}^{2}}{\Phi \left(\mu -2\right)\Gamma \left(3\right)}+\frac{\left(\mu -2\right){\left[G\left(s\right)-G\left(\iota \right)\right]}^{\mu }}{\Phi \left(\mu -2\right)\Gamma \left(\mu +1\right)}]\\ & \;+[\frac{{\left(G\left(\rho \right)-G\left(\iota \right)\right)}^{2}}{\left|Z\right|}+1]\frac{\delta_{1}}{1-\delta_{3}}[\frac{\left(3-\mu \right){\left[G\left(\rho \right)-G\left(\iota \right)\right]}^{2}}{\Phi \left(\mu -2\right)\Gamma \left(3\right)}+\frac{\left(\mu -2\right){\left[G\left(\rho \right)-G\left(\iota \right)\right]}^{\mu }}{\Phi \left(\mu -2\right)\Gamma \left(\mu +1\right)}]\\ & \;+\frac{\left|\xi \right|{\left(G\left(\rho \right)-G\left(\iota \right)\right)}^{2}}{\left|Z\right|}\frac{\delta_{2}}{1-\delta_{3}}[\frac{\left(3-\mu \right){\left[G\left(s\right)-G\left(\iota \right)\right]}^{2}}{\Phi \left(\mu -2\right)\Gamma \left(3\right)}+\frac{\left(\mu -2\right){\left[G\left(s\right)-G\left(\iota \right)\right]}^{\mu }}{\Phi \left(\mu -2\right)\Gamma \left(\mu +1\right)}]\left\|\text{y}\right\|\\ & \;+[\frac{{\left(G\left(\rho \right)-G\left(\iota \right)\right)}^{2}}{\left|Z\right|}+1]\frac{\delta_{2}}{1-\delta_{3}}[\frac{\left(3-\mu \right){\left[G\left(\rho \right)-G\left(\iota \right)\right]}^{2}}{\Phi \left(\mu -2\right)\Gamma \left(3\right)}+\frac{\left(\mu -2\right){\left[G\left(\rho \right)-G\left(\iota \right)\right]}^{\mu }}{\Phi \left(\mu -2\right)\Gamma \left(\mu +1\right)}]\left\|\text{y}\right\|. \end{array}$$

Hence, ‖ℵy‖ ≤ Π_1_ + Π_2_‖y‖ and this finishes the proof.

**Theorem 3.4** *Under assumption* (*AS*_1_) *with* *δ*_3_ ≠ 1, *the mapping* ℵ: Ω → Ω *is compact and consequently is χ-Lipschitz with the Lipschitz’s constant zero*.

**Proof** The boundedness of ℵ implied from Theorem 3.3. It remains to prove that ℵ is an equi-continuous mapping. Therefore, by the assumption (*AS*_1_), for any $\text{y}\in D_{r}$ and *υ*_1_, *υ*_2_ ∈ J with *υ*_1_ < *υ*_2_, we get

|(ℵy)(υ2)-(ℵy)(υ1)|≤|ξ||(G(υ2)-G(ι))2-(G(υ1)-G(ι))2||Z|IABιμ,G|ϖ(s,y(s),DABCιμ,Gy(s))|+|(G(υ2)-G(ι))2-(G(υ1)-G(ι))2||Z|IABιμ,G|ϖ(ρ,y(ρ),DABCιμ,Gy(ρ))|+|IABιμ,Gϖ(υ2,y(υ2),DABCιμ,Gy(υ2))-IABιμ,Gϖ(υ1,y(υ1),DABCιμ,Gy(υ1))|≤|ξ||(G(υ2)-G(ι))2-(G(υ1)-G(ι))2||Z|(δ11-δ3+rδ21-δ3)×[(3-μ)[G(s)-G(ι)]2Φ(μ-2)Γ(3)+(μ-2)[G(s)-G(ι)]μΦ(μ-2)Γ(μ+1)]+|(G(υ2)-G(ι))2-(G(υ1)-G(ι))2||Z|(δ11-δ3+rδ21-δ3)×[(3-μ)[G(ρ)-G(ι)]2Φ(μ-2)Γ(3)+(μ-2)[G(ρ)-G(ι)]μΦ(μ-2)Γ(μ+1)]+(δ11-δ3+rδ21-δ3)|[(3-μ)[G(υ2)-G(ι)]2Φ(μ-2)Γ(3)+(μ-2)[G(υ2)-G(ι)]μΦ(μ-2)Γ(μ+1)]-[(3-μ)[G(υ1)-G(ι)]2Φ(μ-2)Γ(3)+(μ-2)[G(υ1)-G(ι)]μΦ(μ-2)Γ(μ+1)]|.

Obviously, |(ℵy)(*υ*_2_) − (ℵy)(*υ*_1_)| → 0 whenever *υ*_2_ → *υ*_1_ and thus $ℵ\left(D_{r}\right)$ is equi-continuous. Hence, due to Arzelá-Ascoli theorem, $ℵ\left(D_{r}\right)$ is compact and in view of Lemma 2.8, the mapping ℵ is *χ*-Lipschitz with the Lipschitz’s constant *ϵ* = 0.

**Theorem 3.5** *Under assumption* (*AS*_2_), *the* G-ABC **IFDE** (1.3) *possesses an one solution on condition of*

$$\begin{array}{cc} \Pi_{3} & ≔\frac{\left|\xi \right|{\left(G\left(\rho \right)-G\left(\iota \right)\right)}^{2}}{\left|Z\right|}\frac{\ell_{1}}{1-\ell_{2}}[\frac{\left(3-\mu \right){\left[G\left(s\right)-G\left(\iota \right)\right]}^{2}}{\Phi \left(\mu -2\right)\Gamma \left(3\right)}+\frac{\left(\mu -2\right){\left[G\left(s\right)-G\left(\iota \right)\right]}^{\mu }}{\Phi \left(\mu -2\right)\Gamma \left(\mu +1\right)}]\\ & +\left(\frac{{\left(G\left(\rho \right)-G\left(\iota \right)\right)}^{2}}{\left|Z\right|}+1\right)\frac{\ell_{1}}{1-\ell_{2}}[\frac{\left(3-\mu \right){\left[G\left(\rho \right)-G\left(\iota \right)\right]}^{2}}{\Phi \left(\mu -2\right)\Gamma \left(3\right)}+\frac{\left(\mu -2\right){\left[G\left(\rho \right)-G\left(\iota \right)\right]}^{\mu }}{\Phi \left(\mu -2\right)\Gamma \left(\mu +1\right)}]<1. \end{array}$$ (3.6)

**Proof** Let us take the mapping ℵ as given in (3.3). For any $\text{y},\hat{\text{y}}\in \Omega$ and *υ* ∈ J, we find

|(ℵy)(υ)-(ℵy^)(υ)|≤|ξ|(G(υ)-G(ι))2|Z|IABιμ,G|ϖ(s,y(s),DABCιμ,Gy(s))-ϖ(s,y^(s),DABCιμ,Gy^(s))|+(G(υ)-G(ι))2|Z|IABιμ,G|ϖ(ρ,y(ρ),DABCιμ,Gy(ρ))-ϖ(ρ,y^(ρ),DABCιμ,Gy^(ρ))|+IABιμ,G|ϖ(υ,y(υ),DABCιμ,Gy(υ))-ϖ(υ,y^(υ),DABCιμ,Gy^(υ))|≤|ξ|(G(ρ)-G(ι))2|Z|ℓ11-ℓ2[(3-μ)[G(s)-G(ι)]2Φ(μ-2)Γ(3)+(μ-2)[G(s)-G(ι)]μΦ(μ-2)Γ(μ+1)]‖y-y^‖+(G(ρ)-G(ι))2|Z|ℓ11-ℓ2[(3-μ)[G(ρ)-G(ι)]2Φ(μ-2)Γ(3)+(μ-2)[G(ρ)-G(ι)]μΦ(μ-2)Γ(μ+1)]‖y-y^‖+ℓ11-ℓ2[(3-μ)[G(ρ)-G(ι)]2Φ(μ-2)Γ(3)+(μ-2)[G(ρ)-G(ι)]μΦ(μ-2)Γ(μ+1)]‖y-y^‖≤Π3‖y-y^‖.

Thus, $\|ℵ\text{y}-ℵ\hat{\text{y}}\|\leq \Pi_{3}\left\|\text{y}-\hat{\text{y}}\right\|$. Therefore, by condition (3.6), ℵ is contraction mapping and based on the Banach contraction theorem, ℵ possesses a unique fixed point which is a solution of the G-ABC **IFDE** (1.3).

**Theorem 3.6** *Under the assumptions* (*AS*_1_) *and* (*AS*_2_), *the* G-ABC **IFDE** (1.3) *admits a solution such that* Π_2_ < 1. *Furthermore, the set containing solutions of the* G-ABC **IFDE** (1.3) *is bounded*.

**Proof** According Theorem 3.5, ℵ is Lipschitz mapping and by Lemma 2.9, ℵ is *χ*-Lipschitz which implies that ℵ is *χ*-condensing.

Now, due to Theorem 2.10, it remains to show that the set W is bounded, where

$$\begin{array}{c} W=\{\text{y}\in \Omega :\;\text{y}=\zeta ℵ\left(\text{y}\right),\;\text{for}\;\text{some}\;\zeta \in [0,1]\}. \end{array}$$

For end this, let y∈W, therefore for each *υ* ∈ J for some *ζ* ∈ [0, 1], and by Theorem 3.3, we can derive that

$$\begin{array}{c} \left\|\text{y}\right\|=\left\|\zeta \;ℵ\left(\text{y}\right)\right\|\leq \Pi_{1}+\Pi_{2}\left\|\text{y}\right\|. \end{array}$$

Thus, $\left\|\text{y}\right\|\leq \frac{\Pi_{1}}{1-\Pi_{2}},$ which implies that W is a bounded set contained in Ω. In view of Theorem 2.10, implies that ℵ has at least one fixed point, which are act solutions of the G-ABC **IFDE** (1.3), and consequently W contains solutions of the Eq (1.3) is a bounded subset of Ω.

## 4 Stability analysis

Here, we will discuss the stability of UH type. So, we need to state the definitions of UH stability:

**Definition 4.1** [37] *Let there is a real constant Ξ*_*ϖ*_ > 0, *such that for all ς* > 0. *Then the* G-ABC **IFDE** (1.3), *is called* UH *stable when* $\hat{\text{y}}\in \Omega$ *is satisfying the relation*

|DABCιμ,Gy^(υ)-ϖ(υ,y^(υ),DABCιμ,Gy^(υ))|≤ς,υ∈[ι,ρ], (4.1)

*hence there is one function* y ∈ Ω *satisfying the* Eq (1.3), *provided*

$$\begin{array}{c} |\hat{\text{y}}\left(\upsilon \right)-\text{y}\left(\upsilon \right)|\leq \Xi_{\varpi }\varsigma ,\;\upsilon \in \left[\iota ,\rho \right]. \end{array}$$ (4.2)

*Moreover, the solution* y ∈ Ω *of the* Eq (1.3) *is called generalized* UH (GUH) *stable, if there is a function* $\Theta \in C\left(R^{+},R^{+}\right),\Theta \left(0\right)=0$ *satisfied*

$$\begin{array}{c} |\hat{\text{y}}\left(\upsilon \right)-\text{y}\left(\upsilon \right)|\leq \Xi_{\varpi }\Theta \left(\varsigma \right),\;\upsilon \in \left[\iota ,\rho \right]. \end{array}$$ (4.3)

**Remark 4.2** *The function* $\hat{\text{y}}\in \Omega$ *satisfying the inequality* (4.1), *iff there is a function σ* ∈ Ω, *where*

*1)* |*σ*(*υ*)| ≤ ς, *υ* ∈ [*ι*, *ρ*], *ς* > 0;

*2)* DABCιμ,Gy^(υ)=ϖ(υ,y^(υ),DABCιμ,Gy^(υ))+σ(υ) .

**Theorem 4.3** *Let the arguments of Theorem 3.5 are satisfied. Then, the solution of* G-ABC **IFDE** (1.3) *is* UH *and consequently* GUH *stable*.

**Proof** Suppose that $\hat{\text{y}}\in \Omega$ satisfying the Ineq. (4.1), then by applying (4.2), we get

DABCιμ,Gy^(υ)=ϖ(υ,y^(υ),DABCιμ,Gy^(υ))+σ(υ),∀υ∈[ι,ρ].

According to Eq (3.2), one has

y^(υ)=ξ(G(υ)-G(ι))2ZIABιμ,Gϖ(s,y^(s),DABCιμ,Gy^(s))+ξ(G(υ)-G(ι))2ZIABιμ,Gσ(s)-(G(υ)-G(ι))2ZIABιμ,Gϖ(ρ,y^(ρ),DABCιμ,Gy^(ρ))-(G(υ)-G(ι))2ZIABιμ,Gσ(ρ)+IABιμ,Gϖ(υ,y^(υ),DABCιμ,Gy^(υ))+IABιμ,Gσ(υ), (4.4)

which gives

|y^(υ)-ξ(G(υ)-G(ι))2ZIABιμ,Gϖ(s,y^(s),DABCιμ,Gy^(s))+(G(υ)-G(ι))2ZIABιμ,Gϖ(ρ,y^(ρ),DABCιμ,Gy^(ρ))-IABιμ,Gϖ(υ,y^(υ),DABCιμ,Gy^(υ))|≤|ξ|(G(υ)-G(ι))2|Z|IABιμ,G|σ(s)|+(G(υ)-G(ι))2|Z|IABιμ,G|σ(ρ)|+IABιμ,G|σ(υ)|≤|ξ|(G(ρ)-G(ι))2|Z|[(3-μ)[G(s)-G(ι)]2Φ(μ-2)Γ(3)+(μ-2)[G(s)-G(ι)]μΦ(μ-2)Γ(μ+1)]ς+[(G(ρ)-G(ι))2|Z|+1][(3-μ)[G(ρ)-G(ι)]2Φ(μ-2)Γ(3)+(μ-2)[G(ρ)-G(ι)]μΦ(μ-2)Γ(μ+1)]ς. (4.5)

Next, for $\text{y},\hat{\text{y}}\in \Omega$, by utilizing Eqs (4.4) and (4.5) and (*AS*_2_), we have

|y^(υ)-y(υ)|=|y^(υ)-ξ(G(υ)-G(ι))2ZIABιμ,Gϖ(s,y(s),DABCιμ,Gy(s))+(G(υ)-G(ι))2ZIABιμ,Gϖ(ρ,y(ρ),DABCιμ,Gy(ρ))-IABιμ,Gϖ(υ,y(υ),DABCιμ,Gy(υ))|≤|y^(υ)-ξ(G(υ)-G(ι))2ZIABιμ,Gϖ(s,y^(s),DABCιμ,Gy^(s))+(G(υ)-G(ι))2ZIABιμ,Gϖ(ρ,y^(ρ),DABCιμ,Gy^(ρ))-IABιμ,Gϖ(υ,y^(υ),DABCιμ,Gy^(υ))|+|ξ|(G(υ)-G(ι))2|Z|IABιμ,G|ϖ(s,y^(s),DABCιμ,Gy^(s))-ϖ(s,y(s),DABCιμ,Gy(s))|+(G(υ)-G(ι))2|Z|IABιμ,G|ϖ(ρ,y^(ρ),DABCιμ,Gy^(ρ))-ϖ(ρ,y(ρ),DABCιμ,Gy(ρ))|+IABιμ,G|ϖ(υ,y^(υ),DABCιμ,Gy^(υ))-ϖ(υ,y(υ),DABCιμ,Gy(υ))|≤|ξ|(G(ρ)-G(ι))2|Z|[(3-μ)[G(s)-G(ι)]2Φ(μ-2)Γ(3)+(μ-2)[G(s)-G(ι)]μΦ(μ-2)Γ(μ+1)]ς+[(G(ρ)-G(ι))2|Z|+1][(3-μ)[G(ρ)-G(ι)]2Φ(μ-2)Γ(3)+(μ-2)[G(ρ)-G(ι)]μΦ(μ-2)Γ(μ+1)]ς+|ξ|(G(ρ)-G(ι))2|Z|ℓ11-ℓ2[(3-μ)[G(s)-G(ι)]2Φ(μ-2)Γ(3)+(μ-2)[G(s)-G(ι)]μΦ(μ-2)Γ(μ+1)]‖y^-y‖+(G(ρ)-G(ι))2|Z|ℓ11-ℓ2[(3-μ)[G(ρ)-G(ι)]2Φ(μ-2)Γ(3)+(μ-2)[G(ρ)-G(ι)]μΦ(μ-2)Γ(μ+1)]‖y^-y‖+ℓ11-ℓ2[(3-μ)[G(ρ)-G(ι)]2Φ(μ-2)Γ(3)+(μ-2)[G(ρ)-G(ι)]μΦ(μ-2)Γ(μ+1)]‖y^-y‖≤Π4ς+Π3‖y^-y‖,

which further implies

$$\begin{array}{c} \|\hat{\text{y}}-\text{y}\|\leq \frac{\Pi_{4}}{1-\Pi_{3}}\varsigma , \end{array}$$ (4.6)

where

$$\begin{array}{cc} \Pi_{4}: & =\frac{\left|\xi \right|{\left(G\left(\rho \right)-G\left(\iota \right)\right)}^{2}}{\left|Z\right|}[\frac{\left(3-\mu \right){\left[G\left(s\right)-G\left(\iota \right)\right]}^{2}}{\Phi \left(\mu -2\right)\Gamma \left(3\right)}+\frac{\left(\mu -2\right){\left[G\left(s\right)-G\left(\iota \right)\right]}^{\mu }}{\Phi \left(\mu -2\right)\Gamma \left(\mu +1\right)}]\\ & \;+[\frac{{\left(G\left(\rho \right)-G\left(\iota \right)\right)}^{2}}{\left|Z\right|}+1][\frac{\left(3-\mu \right){\left[G\left(\rho \right)-G\left(\iota \right)\right]}^{2}}{\Phi \left(\mu -2\right)\Gamma \left(3\right)}+\frac{\left(\mu -2\right){\left[G\left(\rho \right)-G\left(\iota \right)\right]}^{\mu }}{\Phi \left(\mu -2\right)\Gamma \left(\mu +1\right)}]. \end{array}$$

Thus, yields that

$$\begin{array}{c} \|\hat{\text{y}}-\text{y}\|\leq \Xi_{\varpi }\;\varsigma ;\;\Xi_{\varpi }≔\frac{\Pi_{4}}{1-\Pi_{3}}. \end{array}$$

Hence, the G-ABC fractional implicit differential problem (1.3) is UH stable. In addition, there is a non-decreasing function Θ: (0, ∞) → (0, ∞), Θ(ς)=ς where Θ(0) = 0, so by (4.6), we find

$$\begin{array}{c} \|\hat{\text{y}}-\text{y}\|\leq \Xi_{\varpi }\Theta \left(\varsigma \right). \end{array}$$

Therefore, the G-ABC **IFDE** (1.3) is GUH stable.

## 5 Applications

This section concerns the applications of the essential results using two comprehensive examples with illustrative graphics and tables.

**Example 5.1** *Consider the* G-ABC **IFDE** *as follows*:

{DABCι2.3,Gy(υ)=2υ3+0.3|cos(υ)|υ2+62.cos(y(υ))+e-υυ3+33sin(DABCι2.3,Gy(υ))y(1)=0,[y(1)]G′=0,y(e)=125y(2.3),2.3=s∈(1,e),υ∈J=[1,e]. (5.1)

*Here*, $\mu =2.3,\iota =1,\rho =e,\xi =\frac{1}{25},s=2.3,$ *and*

ϖ(υ,y(υ),DABCιμ,Gy(υ))=2υ3+0.3|cos(υ)|υ2+62.cos(y(υ))+e-υυ3+33sin(DABC12.3,Gy(υ)).

*Thus, we get*

|ϖ(υ,y1(υ),DABC12.3,Gy1(υ))-ϖ(υ,y2(υ),DABC12.3,Gy2(υ))|≤0.0065|y1(υ)-y2(υ)|+0.0131385|DABC12.3,Gy1(υ)-DABC12.3,Gy2(υ)|,

*hence*, *ℓ*_1_ = 0.0065, *ℓ*_2_ = 0.0131385. *So, we have*

$$\begin{array}{cc} \Pi_{3} & ≔\frac{\left|\xi \right|{\left(G\left(\rho \right)-G\left(\iota \right)\right)}^{2}}{\left|Z\right|}\frac{\ell_{1}}{1-\ell_{2}}[\frac{\left(3-\mu \right){\left[G\left(s\right)-G\left(\iota \right)\right]}^{2}}{\Phi \left(\mu -2\right)\Gamma \left(3\right)}+\frac{\left(\mu -2\right){\left[G\left(s\right)-G\left(\iota \right)\right]}^{\mu }}{\Phi \left(\mu -2\right)\Gamma \left(\mu +1\right)}]\\ & \;+\left(\frac{{\left(G\left(\rho \right)-G\left(\iota \right)\right)}^{2}}{\left|Z\right|}+1\right)\frac{\ell_{1}}{1-\ell_{2}}[\frac{\left(3-\mu \right){\left[G\left(\rho \right)-G\left(\iota \right)\right]}^{2}}{\Phi \left(\mu -2\right)\Gamma \left(3\right)}+\frac{\left(\mu -2\right){\left[G\left(\rho \right)-G\left(\iota \right)\right]}^{\mu }}{\Phi \left(\mu -2\right)\Gamma \left(\mu +1\right)}]\\ & \approx \{\begin{array}{c} 0.3029277<1,\;when\;G\left(\upsilon \right)=\upsilon^{2};\\ 0.1501725<1,\;when\;G\left(\upsilon \right)=2^{\upsilon };\\ 0.0268289<1,\;when\;G\left(\upsilon \right)=ln\left(\upsilon^{2}\right). \end{array} \end{array}$$

*Then, according to Theorem 3.5, the* G-ABC **IFDE** (5.1) *has one solution. Furthermore, based on Theorem 4.3 the such solution is* UH *stable with*

$$\begin{array}{c} \Xi_{\varpi }≔\frac{\Pi_{4}}{1-\Pi_{3}}\approx \{\begin{array}{c} 64.979638,\;when\;G\left(\upsilon \right)=\upsilon^{2};\\ 26.422625,\;when\;G\left(\upsilon \right)=2^{\upsilon };\\ 4.1222040,\;when\;G\left(\upsilon \right)=ln\left(\upsilon^{2}\right), \end{array} \end{array}$$

*and consequently is* GUH *stable*.

**Fig 1:**
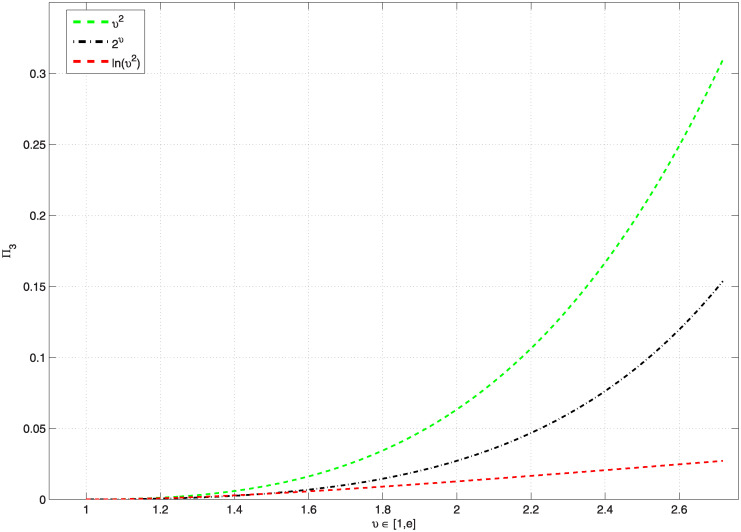
Shows the values of Π_3_ < 1, for various functions G and *υ* ∈ [1, *e*] for problem (5.1).

**Fig 2:**
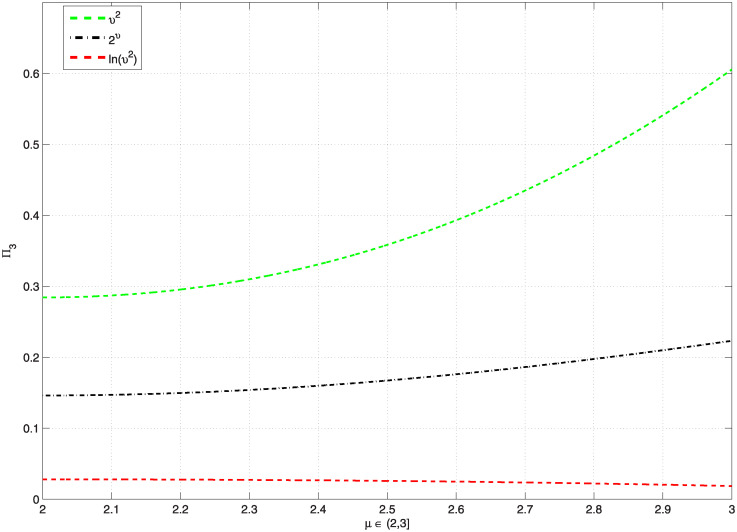
Shows the values of Π_3_ < 1, for some functions G and various *μ* ∈ (2, 3] for problem (5.1).

*Additionally*, Fig 1, *represents the graphics of* Π_3_, *which are less than* 1, *and* Table 1, *shows the computation values of* Π_3_, and Ξ_*ϖ*_, *whenever the function* $G\left(\upsilon \right)=\upsilon^{2},2^{\upsilon },ln\left(\upsilon^{2}\right)$, *on υ* ∈ [1, *e*], *for the problem* (5.1). *Also*, Fig 2, *represents the graphics of* Π_3_, *which are less than 1, and* Table 2, *shows the computation values of* Π_3_ *whenever the function* $G\left(\upsilon \right)=\upsilon^{2},2^{\upsilon },ln\left(\upsilon^{2}\right)$, *and various μ* ∈ (2, 3] *on υ* ∈ [1, *e*] *for problem* (5.1).

**Table 1:** Numerical results of Π_3_ and Ξ_*ϖ*_ at various functions $G\left(\upsilon \right)=\upsilon^{2}$, $G\left(\upsilon \right)=2^{\upsilon }$ and $G\left(\upsilon \right)=ln\left(\upsilon^{2}\right)$ on [1, *e*] for problem 5.1.

*υ*	$G\left(\upsilon \right)=\upsilon^{2}$		$G\left(\upsilon \right)=2^{\upsilon }$		$G\left(\upsilon \right)=ln\left(\upsilon^{2}\right)$	
	Π_3_ < 1	Ξ_*ϖ*_	Π_3_ < 1	Ξ_*ϖ*_	Π_3_ < 1	Ξ_*ϖ*_
1.0000	0.0000	0.0000	0.0000	0.0000	0.0000	0.0000
1.12450	0.0019	0.2800	0.0008	0.1237	0.0012	0.1752
1.4900	0.0097	1.4678	0.0041	0.6227	0.0040	0.6047
1.7350	0.0275	4.2231	0.0116	1.7557	0.0079	1.1861
1.9800	0.0599	9.5295	0.0256	3.9348	0.0123	1.8612
2.2250	0.1128	19.0147	0.0499	7.8485	0.0171	2.5959
2.4700	0.1929	35.7502	0.0896	14.7260	0.0220	3.3688
2.7150	0.3081	66.5950	0.1529	26.9840	0.0271	4.1664

**Table 2:** Numerical results of Π_3_ at various *μ* ∈ (2, 3] and some functions G(υ) on [1, *e*] for problem 5.1.

*μ*	2.1	2.2	2.3	2.4	2.5	2.6	2.7	2.8	2.9	3
Π_3_ at $G\left(\upsilon \right)=\upsilon^{2}$	0.2870	0.2955	0.3099	0.3308	0.3584	0.3930	0.4349	0.4842	0.5410	0.6055
Π_3_ at $G\left(\upsilon \right)=2^{\upsilon }$	0.1470	0.1496	0.1539	0.1598	0.1672	0.1761	0.1862	0.1976	0.2099	0.2232
Π_3_ at $G\left(\upsilon \right)=ln\left(\upsilon^{2}\right)$	0.0278	0.0276	0.0272	0.0266	0.0258	0.0248	0.0236	0.0221	0.0204	0.0186

**Example 5.2** *Consider the* G-ABC **IFDE** *as follows*:

{DABC12.8,Gy(υ)=e-2υ92+e-2υ.11+|y(υ)|+|DABC12.8,Gy(υ)|+e-3υ72DABC12.8,Gy(υ),y(0)=0,[y(0)]G′=0,y(2)=115y(1.3),1.3=s∈(1,2),υ∈J=[1,2], (5.2)

*where*, $\mu =2.8,\iota =1,\rho =2,s=1.3,\xi =\frac{1}{15},$ *and*

ϖ(υ,y(υ),DABC12.8,Gy(υ))=e-2υ92+e-2υ.11+|y(υ)|+|DABC12.8,Gy(υ)|+e-3υ72DABC12.8,Gy(υ).

*Thus, we get*

|ϖ(υ,y1(υ),DABCιμ,Gy1(υ))-ϖ(υ,y2(υ),DABCιμ,Gy2(υ))|≤0.0123456|y1(υ)-y2(υ)|+0.0327538|DABC12.8,Gy1(υ)-DABC12.8,Gy2(υ)|,

*hence, ℓ*_1_ = 0.0123456, *ℓ*_2_ = 0.0327538. *So, we have*

$$\begin{array}{cc} \Pi_{3} & \approx \{\begin{array}{c} 0.3821305<1,\;when\;G\left(\upsilon \right)=e^{\upsilon };\\ 0.8552526<1,\;when\;G\left(\upsilon \right)=0.9\upsilon^{3};\\ 0.1590327<1,\;when\;G\left(\upsilon \right)=\text{sin}\left(\frac{\upsilon }{2}\right)+\upsilon^{2}. \end{array} \end{array}$$

*Then, in view of Theorem 3.5, the* G-ABC **IFDE** (5.2) *has one solution. Furthermore, based on Theorem 4.3 the such solution is* UH *stable with*

$$\begin{array}{c} \Xi_{\varpi }≔\frac{\Pi_{4}}{1-\Pi_{3}}\approx \{\begin{array}{c} 48.454820,\;when\;G\left(\upsilon \right)=e^{\upsilon };\\ 400.62919,\;when\;G\left(\upsilon \right)=0.9\upsilon^{3};\\ 14.815947,\;when\;G\left(\upsilon \right)=\text{sin}\left(\frac{\upsilon }{2}\right)+\upsilon^{2}, \end{array} \end{array}$$

*and consequently is* GUH *stable*.

**Fig 3:**
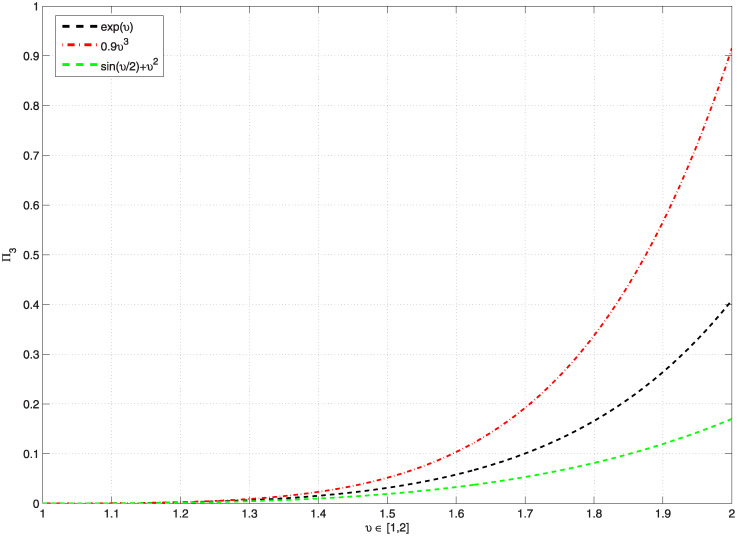
Shows the values of Π_3_ < 1, for various functions G and *υ* ∈ [1, 2] of the problem (5.2).

**Fig 4:**
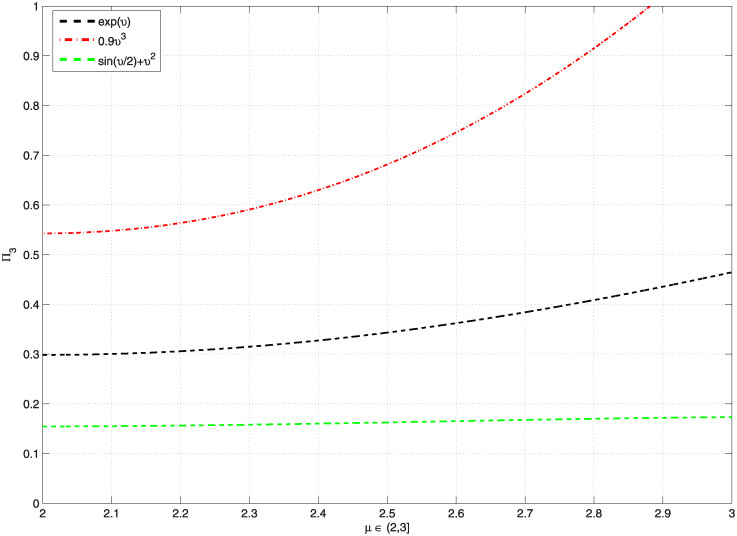
Shows the values of Π_3_ < 1, for some functions G and various *μ* ∈ (2, 3] of problem (5.2).

*Moreover*, Fig 3, *represents the graphics of* Π_3_, *which are less than* 1, *and* Table 3, *shows the computation values of* Π_3_, *and* Ξ_*ϖ*_, *whenever the function* $G\left(\upsilon \right)=e^{\upsilon },\;0.9\upsilon^{3},\;\text{sin}\left(\frac{\upsilon }{2}\right)+\upsilon^{2}$, *on υ* ∈ [1, 2], *for the problem* (5.2). *In addition*, Fig 4, *represents the graphics of* Π_3_, *which are less than* 1, *and* Table 4, *shows the computation values of* Π_3_ *whenever the function* $G\left(\upsilon \right)=e^{\upsilon },\;0.9\upsilon^{3},\;\text{sin}\left(\frac{\upsilon }{2}\right)+\upsilon^{2}$, *and various μ* ∈ (2, 3] *on υ* ∈ [1, 2] *for problem* (5.2). *According to* Fig 4 *and* Table 4, *we observe that* Π_3_ ≥ 1 *for some values μ at function* $G\left(\upsilon \right)=0.9\upsilon^{3}$, *thus for this reason and only at these values we can’t say that the problem* (5.2) *has one solution*.

**Table 3:** Numerical results of Π_3_ and Ξ_*ϖ*_ of some functions $G\left(\upsilon \right)=e^{\upsilon }$, $G\left(\upsilon \right)=0.9\upsilon^{3}$, and $G\left(\upsilon \right)=\text{sin}\left(\frac{\upsilon }{2}\right)+\upsilon^{2}$, on [1, 2] for problem 5.2.

*υ*	$G\left(\upsilon \right)=e^{\upsilon }$		$G\left(\upsilon \right)=0.9\upsilon^{3}$		$G\left(\upsilon \right)=\text{sin}\left(\frac{\upsilon }{2}\right)+\upsilon^{2}$	
	Π_3_ < 1	Ξ_*ϖ*_	Π_3_ < 1	Ξ_*ϖ*_	Π_3_ < 1	Ξ_*ϖ*_
1.0000	0.0000	0.0000	0.0000	0.0000	0.0000	0.0000
1.1250	0.0006	0.0493	0.0007	0.0558	0.0005	0.0369
1.2500	0.0039	0.3059	0.0050	0.3974	0.0027	0.2143
1.3750	0.0126	1.0026	0.0186	1.4848	0.0083	0.6538
1.5000	0.0313	2.5335	0.0516	4.2628	0.0190	1.5188
1.6250	0.0668	5.6122	0.1215	10.8329	0.0373	3.0384
1.7500	0.1297	11.6766	0.2563	26.9974	0.0661	5.5489
1.8750	0.2358	24.1681	0.4993	78.1367	0.1089	9.5792
2.0000	0.4086	54.1394	0.9152	845.0222	0.1699	16.0365

**Table 4:** Numerical results of Π_3_ at various *μ* ∈ (2, 3] and some functions G(υ) on [1, 2] for problem 5.2.

*μ*	2.1	2.2	2.3	2.4	2.5	2.6	2.7	2.8	2.9	3
Π_3_ at $G\left(\upsilon \right)=e^{\upsilon }$	0.3002	0.3057	0.3148	0.3273	0.3432	0.3622	0.3841	0.4086	0.4355	0.4645
Π_3_ at $G\left(\upsilon \right)=0.9\upsilon^{3}$	0.5479	0.5636	0.5908	0.6298	0.6813	0.7458	0.8237	0.9152	1.0205	1.1398
Π_3_ at $G\left(\upsilon \right)=\text{sin}\left(\frac{\upsilon }{2}\right)+\upsilon^{2}$	0.1550	0.1562	0.1579	0.1601	0.1626	0.1651	0.1676	0.1699	0.1718	0.1732

## 6 Conclusions

This manuscript dealt with a new class of G-ABC-**IFDE** (1.3) with higher orders belonging to the interval (2, 3]. The fundamental conditions of the existence and uniqueness of the solution for Eq (1.3) were established by Banach and topology degree theories. Moreover, the UH stability with its generalized was discussed. Finally, two application examples with illustrative graphics and tables were provided to check the effectiveness of the main results with compare the main parameters.

The results of this study can be employed in new problems as special cases of the main Eq (1.3) by taking various functions of G. Furthermore, the GABC-**IFDE** (1.3) covers some problems are existing in the literature; for instance (**i**) the Eq (1.3) can be reduced to problem (1.1) if *μ* → 3 and the implicit term omitted; **(ii)** the Eq (1.3) can be returned to problem (1.2) if we replace the operator DABCιμ,G by DCιμ with omitting the implicit term.
